# Pathogenicity of the Canadian Delmarva (DMV/1639) Infectious Bronchitis Virus (IBV) on Female Reproductive Tract of Chickens

**DOI:** 10.3390/v13122488

**Published:** 2021-12-11

**Authors:** Mohamed S. H. Hassan, Ahmed Ali, Sabrina M. Buharideen, Dayna Goldsmith, Carla S. Coffin, Susan C. Cork, Frank van der Meer, Martine Boulianne, Mohamed Faizal Abdul-Careem

**Affiliations:** 1 Health Research Innovation Center 2C53, Faculty of Veterinary Medicine, University of Calgary, 3330 Hospital Drive NW, Calgary, AB T2N 4N1, Canada; msh.hassan@ucalgary.ca (M.S.H.H.); Ahmed.Ali@ucalgary.ca (A.A.); sabrina.buharideen@ucalgary.ca (S.M.B.); dayna.goldsmith@ucalgary.ca (D.G.); sccork@ucalgary.ca (S.C.C.); fjvander@ucalgary.ca (F.v.d.M.); 2 Department of Poultry Diseases, Faculty of Veterinary Medicine, Assiut University, Assiut 71515, Egypt; 3 Department of Pathology, Faculty of Veterinary Medicine, Beni-Suef University, Beni Suef 62521, Egypt; 4 Health Research Innovation Center 2C52, Department of Medicine, Cumming School of Medicine, University of Calgary, 3330 Hospital Drive NW, Calgary, AB T2N 4N1, Canada; cscoffin@ucalgary.ca; 5 Department of Clinical Sciences, Faculty of Veterinary Medicine, University of Montréal, St. Hyacinthe, QC J2S 2M2, Canada; martine.boulianne@umontreal.ca

**Keywords:** infectious bronchitis virus (IBV), Delmarva (DMV)/1639 strain, white leghorn chicken, cystic oviduct, false layer syndrome

## Abstract

Infectious bronchitis virus (IBV) infection causes significant economic losses to various sectors of the poultry industry worldwide. Over the past few years, the incidence of false layer syndrome in Eastern Canadian layer flocks has been associated with the increased prevalence of the IBV Delmarva (DMV)/1639 strain. In this study, 1-day-old specific-pathogen-free (SPF) hens were infected with the Canadian DMV/1639 strain and observed until 16 weeks of age in order to determine if the IBV DMV/1639 strain is causing false layer syndrome. Early after infection, the virus showed a wide tissue distribution with characteristic gross and histopathological lesions in the respiratory tract and kidney. Around 60–70% of the infected hens demonstrated continuous cloacal viral shedding until the end of the experiment (at 16 weeks) which was associated with high IBV genome loads detected in the cecal tonsils. The experiment confirmed the field observations that the Canadian DMV/1639 strain is highly pathogenic to the female reproductive tract causing marked cystic lesions in the oviduct. Moreover, significant histopathological damage was observed in the ovary. Our study provides a detailed description of the pathological consequences of the IBV DMV/1639 strain circulating in an important poultry production sector.

## 1. Introduction

Infectious bronchitis (IB) is currently endemic and poses an economic threat to most countries with large-scale poultry production [1,2]. The causative agent, infectious bronchitis virus (IBV), is a gammacoronavirus of the family *Coronaviridae*. IBV is primarily a respiratory pathogen where initial infection and replication occur within the epithelial layer of the upper respiratory tract of chickens causing signs such as gasping, coughing, sneezing, tracheal rales, and nasal discharge [3]. Infected chickens usually develop serous, catarrhal, or caseous exudate covering the thickened mucosa of the trachea, nasal passages, and sinuses. Areas of pneumonia have also been observed in the lungs [4]. IBV also targets lung macrophages [5] and monocytes [6] and is believed to spread beyond the respiratory tract via lymph and/or blood [7]. In addition to the respiratory form of the disease, IBV infection is also associated with renal, reproductive, and gastrointestinal pathologies depending on the infecting IBV strain [8]. The emergence of genetically diverse IBV strains is associated with rapid mutation rates, viral recombination, and selection processes [9,10]. Despite the early introduction of live attenuated vaccines to control IBV infection [11], the poor cross protection between heterologous strains compromises efforts to prevent the disease [12].

Nephropathogenicity is mostly described in broilers. Several QX-type IBV strains, identified throughout Asia, Europe, and Africa, are associated with renal pathology [13,14,15]. Gray, PA/Wolgemuth/98, PA/171/99, 98, and DMV/1639/11 are among the nephropathogenic IBV strains found in the United States of America (USA) [16,17,18]. In Australia, the T strain was responsible for some of the most severe nephropathogenic IB (NIB) outbreaks [19]. Nephropathogenic IBV strains cause nephritis leading to gross lesions such as swollen and pale kidneys with distended tubules and ureters due to urate accumulation [4,20]. Infected flocks show increased water intake, watery dropping, weight loss, and increased mortality [17].

Infection of the reproductive tract by certain IBV strains has been associated with adverse effects on egg production [8]. Over the years, infection of laying hens with the Massachusetts (Mass) type of IBV has led to decline in egg production and disorders of egg formation [21,22]. Hens infected with Australian T strain showed long-term loss of internal egg quality [23]. IBV infection of young female chicks can cause permanent damage to the developing reproductive tracts resulting in cystic oviducts. Although chickens with cystic oviducts appear healthy, they are unable to lay eggs, and are commonly referred to as false layers leading to culling of affected flocks [24]. The cystic oviduct lesions have been reported in chickens infected with Australian T, Mass, and QX strains before 3 weeks of age [24,25,26,27]. The incidence of cystic oviduct following IBV infection tends to decrease with increasing age at first exposure [28,29].

In 2011, a new IBV variant designated as DMV/1639 was associated with multiple NIB outbreaks in the Delmarva peninsula, USA [18]. Since 2015, IBV strains that are genetically identical to the DMV/1639 variant started to be dominant in poultry operations in Eastern Canada [30,31]. These IBV strains were isolated from apparently healthy laying flocks presenting with poor egg production (peak of lay ranging from 40 to 77%) in Ontario and Quebec. At necropsy, cystic left oviducts have been observed suggesting false layers [32,33]. Similarly, the DMV/1639 IBV strain was also isolated from a breeder flock in Pennsylvania, USA that showed segmented cystic left oviduct during necropsy examinations [34]. These observations suggest that infection with the DMV/1639 strain is associated with the incidence of false layers. This study aimed to investigate the pathogenicity and tissue tropism of the Canadian DMV/1639 strain in hens challenged at 1-day of age.

## 2. Materials and Methods

### 2.1. Virus

The Canadian IBV DMV/1639 strain used in this study, designated as IBV/Ck/Can/17-036989, was described previously [31]. This strain was isolated in 2017 from a layer flock in Ontario, Canada with a history of decreased egg production. The fourth passage, in embryonated chicken eggs (ECE), of the virus was titrated by inoculating 10-day-old specific-pathogen-free (SPF) embryonated eggs with 10-fold serial dilutions of the virus stock (allantoic fluid) via the chorioallantoic sac. The inoculated eggs were observed for 7 days post-inoculation (dpi) for embryonic deaths and lesions characteristic for IBV infection (stunting and curling). The 50% chicken embryo infectious dose (EID_50_) was calculated with the method of Reed and Muench [35]. The titer of the virus stock was determined to be 1 × 10^7^ EID_50_/ mL and stored as aliquots of 0.1 mL allantoic fluid at −80 °C.

### 2.2. Chickens

White Leghorn SPF eggs, obtained from the Canadian Food Inspection Agency (CFIA), Ottawa, Ontario, were incubated and hatched in our laboratory, Foothills campus, University of Calgary. Hatching chicks were sexed, and day-old SPF females (n = 40) were housed in the Veterinary Science Research Station (VSRS) facility at the Spy Hill campus, University of Calgary. The feed and lightening system were adjusted according to the management guidelines recommended for White Leghorn growing pullets.

### 2.3. Experimental Procedures

#### 2.3.1. Grouping and Viral Challenge

Day-old chicks were randomly divided into two groups (20 chicks in each) and housed in two separate negative pressure rooms. The chicks in the first group were infected with 100 μL of the challenge virus containing 1 × 10^6^ EID_50_ by the oculo-nasal route [36]. The chicks in the second group were mock inoculated with equal volume of phosphate buffered saline (PBS).

#### 2.3.2. Clinical Observations and Swab Collection

Birds in both groups were monitored twice daily for clinical signs, which were scored ranging from 0 to 3 as described previously [37]. Briefly, general clinical manifestations such as ruffled feathers and huddling under a heat source, depression with lowered head, dropped wings, and diarrhea received a score of 1. Respiratory signs were scored 1 if mild (increased respiration but beaks remained closed), 2 if moderate (increased respiration with open beaks, coughing, sneezing, watery eyes, and nasal discharge), and 3 if severe (marked gasping). A score of 0 was given in case of the absence of any of the previously mentioned clinical signs. A bird that reaches a cumulative score of 5 was euthanized immediately on humane grounds.

Oropharyngeal (OP) and cloacal (CL) swabs were collected weekly from all birds in both groups using the Puritan^®^ Unitranz-RT^®^ Transport System (Puritan Medical Products Co., Guilford, ME, USA). Collected swabs were vortexed and aliquots of 250 µL of the transport medium were stored at −80 °C until processing.

#### 2.3.3. Postmortem Examination and Sample Collection

At 7 dpi, under isoflurane anesthesia, 1 mL of intracardiac blood was collected from four chicks of the infected group and five chicks of the control group followed by euthanasia through cervical dislocation. Portions of trachea, lung, kidney, ovary, oviduct, and cecal tonsils (CT) were collected in RNA Save^®^ (Biological Industries, Beit Haemek, Israel) and Optimum Cutting Temperature (OCT) compound (VWR International, Edmonton, AB, Canada). Additionally, portions of trachea, lung, and kidney were collected in 10% neutral buffered formalin (VWR International, Edmonton, AB, Canada). At 14, 21, 28, 35, and 91 dpi, l ml of wing vein blood was collected from all birds in both groups.

At 112 dpi (16 weeks of age), all the remaining birds in both groups were euthanized by cervical dislocation under isoflurane anesthesia. Portions of kidney, ovary, oviduct, and cecal tonsils were collected in RNA Save. In addition, portions of previously mentioned tissues, except cecal tonsil, were fixed in 10% neutral buffered formalin.

Tissues collected in RNA Save and OCT compound were preserved at −80 °C until processing. The gross anatomy of the birds that died or were euthanatized during the experiment was examined and recorded.

#### 2.3.4. Assessment of the Development of the Ovary and Oviduct

To evaluate the developmental status of the reproductive systems in both groups, the weights of the ovaries and the lengths of oviducts were measured. The gross cystic lesions of the reproductive tract were estimated and scored using a four-grade-scoring: negative (no cyst), moderate (cyst covering less than 20% of oviduct; +), marked (cyst covering 20% to 50%; ++), and severe (cyst covering more than 50% of oviduct; +++) [38].

### 2.4. Techniques

#### 2.4.1. IBV Genome Load Quantification

Total RNA was extracted from collected swabs and tissues using Trizol^®^ reagent (Invitrogen Canada Inc., Burlington, ON, Canada) following manufacturer’s guidelines. The concentration and quality of the extracted RNA was determined using a Nanodrop1000 spectrophotometer (ThermoScientific, Wilmington, DE, USA), with the absorbance at a 260/280 nm wavelength. One µg RNA of each swab sample and 2 µg RNA of each tissue sample were used to synthesize cDNA with the use of High-Capacity cDNA Reverse Transcription Kit (Invitrogen Life Technologies, Carlsbad, CA, USA) according to manufacturer’s guidelines.

For IBV genome load determination in swab and tissue samples, a SYBR green-based qRT-PCR using primers targeting a conserved region within the IBV nucleocapsid gene (N) was used as previously described [39]. All cDNA preparations were analyzed alongside a dilution series of an in-house prepared plasmid used to generate a standard curve. The assay was performed using Fast SYBR^®^ Green Master Mix (Quntabio^®^, Beverly, MA, USA) in a reaction volume of 20 µL. Each reaction volume contained 10 µL of SYBR Green master mix, 100 ng of respective sample template, 0.5 µL of forward and reverse primers, and DNAse/RNAse-free water. Cycling conditions were 95 °C for 20 s; followed by 40 cycles of amplification/extension at 95 °C for 3 s, and 60 °C for 30 s; melting curve analysis was done at 95 °C for 10 s (Segment 1), 65 °C for 5 s (Segment 2) and 9 °C for 5 s (Segment 3). Fluorescent acquisition was done at 60 °C for 30 s.

#### 2.4.2. Immunofluorescence Assay

Immunofluorescence staining was performed to detect IBV antigen. The tissues preserved in OCT compound were sectioned at 5 μm thickness using a freezing microtome (Leica CM 1850, Leica Biosystems, Buffalo Grove, IL, USA) and adhered on to positively charged slides. The frozen sections were fixed in pre-cooled acetone for 5 min followed by air drying at room temperature for 10 min. Fixed cryosections were treated with 0.2% Triton X-100 (Sigma-Aldrich, Saint Louis, MO, USA) for permeabilization. Blocking was done using 2.5% horse serum diluted in Trizma buffered saline (TBS) at room temperature for 1 h followed by incubation with a mouse monoclonal antibody against the nucleoprotein of IBV (Novus Biologicals, Toronto, ON, Canada) for 30 min (1:400 dilution in blocking buffer). The tissue sections were then covered with amplifier antibody (goat anti-mouse antibody) from the VectaFluor^™^ Excel R.T.U. antibody kit Dylight 594 (Vector Laboratories, Burlingame, CA, USA) and incubated for 15 min followed by 30 min incubation with VectaFluor^™^ Dylight^®^ Dye-labeled anti-goat IgG made in horse serum (VectaFluor™ Excel R.T.U. antibody kit Dylight 594, Vector Laboratories, Burlingame, CA, USA). Finally, coverslips were mounted on glass slides with Vectashield^®^ antifade mounting medium with 4′,6-Diamidine-2′-phenylindole dihydrochloride (DAPI) nuclear stain (Vector Laboratories, Burlingame, CA, USA) and sealed with lacquer. The stained slides were examined using epifluorescent microscopy (Olympus BX51, Center Valley, PA, USA).

#### 2.4.3. Enzyme-Linked Immunosorbent Assay (ELISA)

Commercial ELISA kit (IDEXX Laboratories, Inc., Westbrook, ME, USA) was used to measure the antibody-mediated immune response against IBV in serum (1:500 dilution). The assay was performed according to the manufacturer’s protocol. The antibody titers were calculated with the formula provided by IDEXX, and titers >396 (cut-off) were considered positive.

#### 2.4.4. Histopathology

Tissue samples collected at 7 and 112 dpi and preserved in 10% neutral buffered formalin were embedded in paraffin, sectioned at 5 μm, stained with hematoxylin and eosin (H&E) (Diagnostic Services Unit (DSU) at the University of Calgary), and examined with light microscopy (Olympus BX51, Center Valley, PA, USA) for lesions attributable to IBV infection.

#### 2.4.5. Statistical Analysis

A Mixed-effects model followed by Sidak’s multiple comparisons test was used to identify the differences in clinical scores and anti-IBV antibody titers between infected and control groups and differences in IBV genome loads in the OP and CL swabs over different time points in the infected group. Friedman test followed by Dunn’s multiple comparisons test was used to identify differences in the IBV genome loads in the different tissues of the infected group in each time point. Differences in lengths of oviducts and weights of ovaries were analyzed using Mann–Whitney U test. The frequencies of lesions on oviduct were compared with the Fisher’s exact test. GraphPad Prism 9.2.0 Software (GraphPad Software, San Diego, CA, USA) was used for the data analysis and to generate graphs.

## 3. Results

### 3.1. Clinical Findings

**Figure 1:**
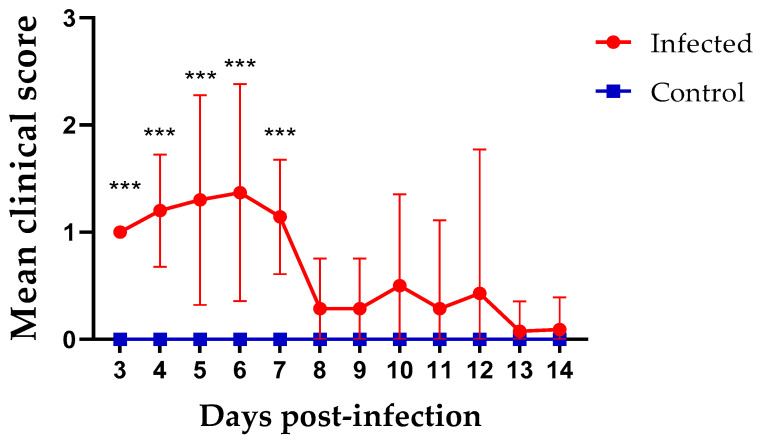
Mean clinical scores for infected and control groups starting from 3 to 14 dpi following infection at 1-day of age. The daily mean clinical scores were compared using Mixed-effects model followed by Sidak’s multiple comparisons test, and the error bars represent the standard deviation (SD). Statistical significance: *** *p* < 0.001.

No birds reached end points and no clinical signs were observed in the control group during the experiment. Chickens in the infected group started to show clinical signs at 3 dpi. The mean clinical scores of the infected group were significantly higher between 3 to 7 dpi compared to the control group (*p* < 0.05). The peak of the clinical signs was recorded at 6 dpi (Figure 1). Most of the birds recovered at 8 dpi; however, only one to four birds continued to show mild respiratory signs until 14 dpi. Excluding birds euthanized for sampling at 7dpi, approximately 19% of the infected chickens were euthanized between 5 and 12 dpi.

### 3.2. Viral Shedding

**Figure 2:**
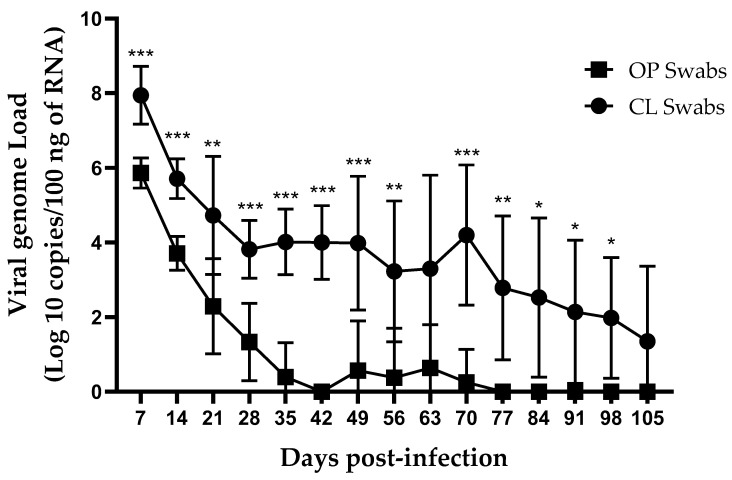
IBV genome loads in weekly collected OP and CL swabs following infection at 1-day of age. The average starting IBV copies was quantified per 100 ng of the extracted RNA and differences between OP and CL swabs were compared using Mixed-effects model followed by Sidak’s multiple comparisons test, and the error bars represent the SD. Statistical significance: * *p* < 0.05, ** *p* < 0.01, *** *p* < 0.001.

No IBV genome loads were detected in the control group during the experiment. In the infected group, the IBV genome loads were quantifiable from OP and CL swabs in approximately 60–70% of birds until 28 dpi and 98 dpi, respectively. Afterward, viral shedding through OP route showed one or two different positive birds. The IBV genome loads in cloacal swabs were significantly higher (*p* < 0.05) than OP swabs at all time points except at 63 and 105 dpi (Figure 2).

### 3.3. Serology

No anti-IBV antibody titer could be detected in the birds in the control group. In the infected group, 23–30% of the birds had positive serum antibody titers between 21 and 35 dpi. At 91 dpi, around 54% of birds showed positive titers. Positive mean titers in the infected group were 482 and 610 at 35 and 91 dpi, respectively. However, the mean titer in the infected group was significantly higher than the control group at 91 dpi only (*p* < 0.05).

### 3.4. IBV Genome Load in Tissues

**Figure 3:**
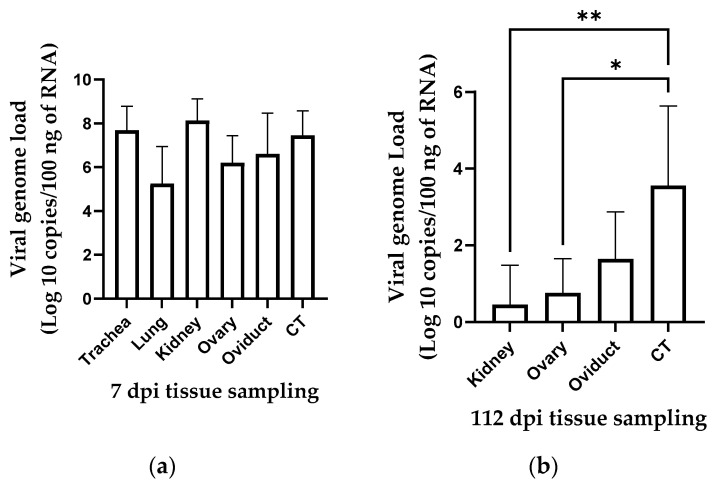
IBV genome loads in different tissue samples collected at 7 dpi (**a**) and 112 dpi (**b**) following infection at 1-day of age. The average starting IBV copies was quantified per 100 ng of the extracted RNA and differences between tissue samples at both time points were compared using Friedman test followed by Dunn’s multiple comparisons test, and the error bars represent the SD. Statistical significance: * *p* < 0.05, ** *p* < 0.01.

No IBV genome loads could be detected in tissues of the control group collected at 7 and 112 dpi. In the infected group, the IBV genome load was quantifiable in all tissues collected at 7 dpi; however, the difference in the genome loads between different tissues was not statistically significant (*p* > 0.05; Figure 3a). At 112 dpi, the highest genome load was detected in the cecal tonsils that was significantly higher than the genome loads detected in the kidney and ovary (*p* < 0.01 and <0.05, respectively; Figure 3b).

### 3.5. Immunofluorescence Staining

**Figure 4:**
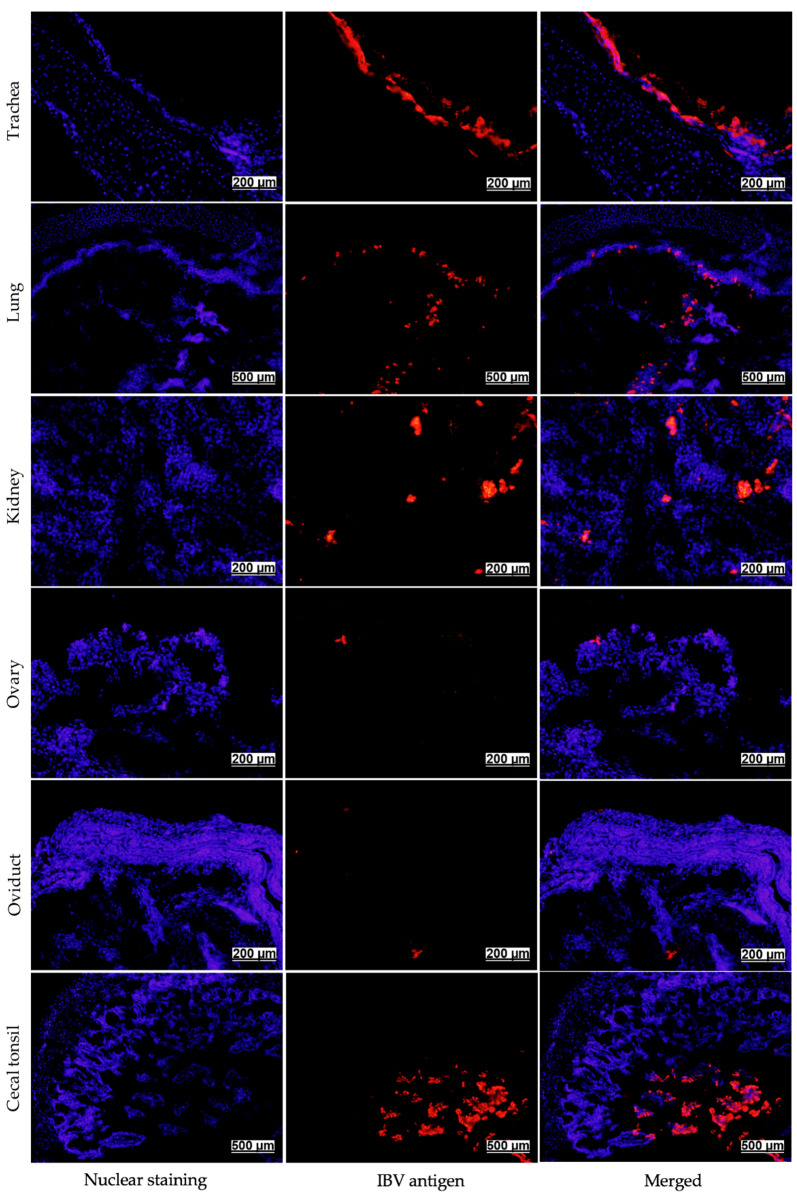
Immunofluorescence assay showing IBV antigens that were visualized with Dylight 594^®^ (Red) in infected trachea, lung, kidney, ovary, oviduct, and cecal tonsil at 7 dpi following infection at 1-day of age. The nuclei were visualized using 4′,6-diamidino-2-phenylindole (DAPI) (blue).

Immunofluorescence staining was performed on tissues to confirm the presence of the IBV antigen. Viral antigen was detected in the epithelium of the infected trachea, lung, kidney, ovary, oviduct, and cecal tonsil collected at 7 dpi (Figure 4).

### 3.6. Gross Pathology and Development of the Reproductive Tract

**Figure 5:**
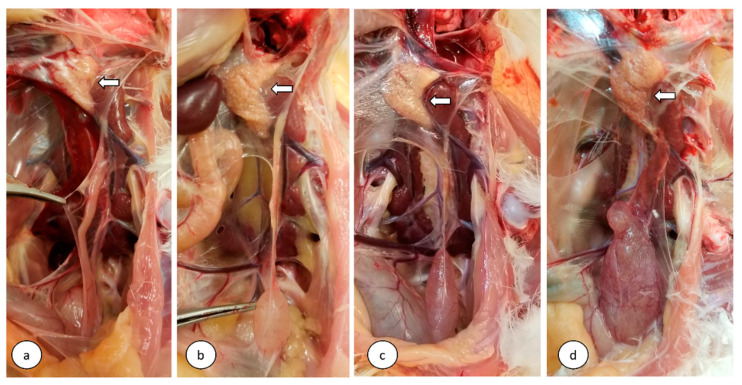
Comparison of the cystic lesions in the oviduct at 112 dpi (16 weeks) following infection at 1-day of age. (**a**) Control normal oviduct, (**b**) moderate cystic lesion, (**c**) marked cystic lesion, and (**d**) severe cystic lesion. Open arrows indicate the immature ovary.

No gross lesions were observed in the control group at 7 and 112 dpi. In the infected group, slight hyperemia of the tracheal mucosa and characteristic pale swollen kidneys with urate deposition in the tubules and ureters were seen in the birds euthanized between 5 and 12 dpi. At 112 dpi (16 weeks of age), 46% of the infected birds showed cystic lesions of varying sizes in the oviduct. The frequency of developing marked cystic lesions in the infected birds was significantly higher compared to the control birds (*p* < 0.05). Additionally, the number of birds with healthy oviducts in the infected group was significantly lower compared to the control group (*p* < 0.05) (Table 1). Cystic lesions were filled with serous clear fluid and were mostly localized in the caudal portion of the undifferentiated oviduct (Figure 5). There were no statistically significant differences in the weights of ovaries and lengths of oviducts between the infected and control groups.

### 3.7. Histopathological Findings

#### 3.7.1. Histopathological Findings at 7 dpi

**Figure 6:**
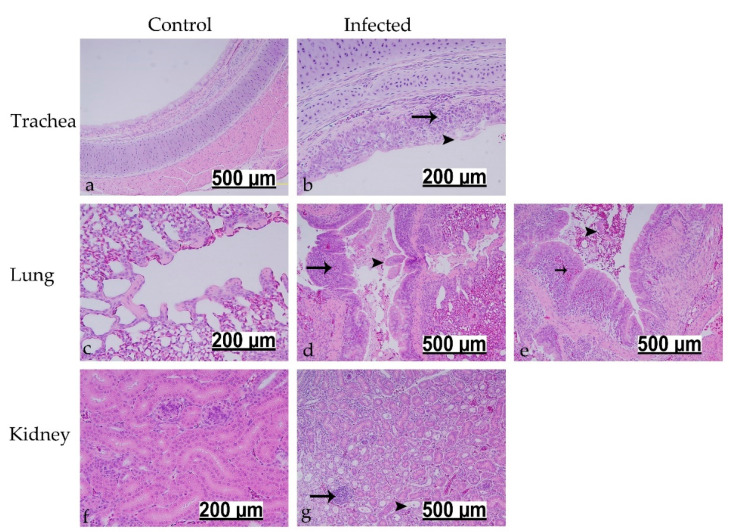
Histopathological changes detected in trachea, lung, and kidney at 7 dpi following infection at 1-day of age. (**a,c,f**) are controls. (**b**) Black arrowhead indicates epithelial desquamation and deciliation; black arrow shows mononuclear cells. (**d**,**e**) Black arrowheads refer to cellular exudate; long black arrow shows lymphoid aggregates; short black arrow reveals heterophilic infiltration. (**g**) Black arrow indicates interstitial focal lymphoplasmacytic cells; black arrowhead shows dilated renal tubules.

The microscopic examination showed no significant pathological changes in the control group at 7 dpi (Figure 6a,c,f). The microscopic lesions in the infected birds were prominent in the trachea (4/5 birds), lung (3/5 birds), and kidney (5/5 birds). The trachea showed epithelial cell necrosis with desquamation and ciliary loss. The lamina propria was thickened because of mononuclear cell infiltrations (Figure 6b). In the lung, the secondary bronchi exhibited hyperplasia of the epithelial lining with cellular exudate (desquamated epithelium and erythrocytes) in the lumen. The lamina propria was infiltrated either by mononuclear cells or heterophils (Figure 6d,e). The kidney revealed lymphoplasmacytic interstitial nephritis, which was recognized by focal to multi-focal mononuclear cell infiltrations accompanied by dilatation of some renal tubules (Figure 6g). The renal tubular epithelium showed necrotic changes in the form of pyknotic nuclei and hypereosinophilic cellular debris.

#### 3.7.2. Histopathological Findings at 112 dpi

**Figure 7:**
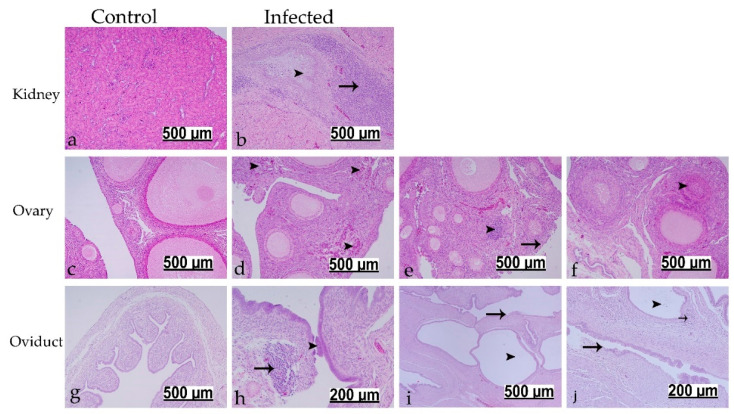
Histopathological changes detected in kidney, ovary, and oviduct at 112 dpi following infection at 1-day of age. (**a**,**c**,**g**) are controls. (**b**) Black arrowhead indicates intra-luminal mucus accumulations; black arrow shows massive mononuclear cells at lamina propria. (**d**) Black arrowheads show multi-focal heterophilic infiltrations. (**e**) Black arrowhead refers to focal mononuclear cell infiltration; black arrow refers to necrosis and sloughing of ovarian covering epithelium. (**f**) Black arrowhead indicates follicle undergoes obliterative follicular atresia. (**h**) Black arrowhead refers to sloughed epithelium; black arrow shows focal mononuclear cell aggregates. (**i**,**j**) Black arrowheads reveal cystic dilated glands; black arrows show sloughed epithelium; short black arrow refers to mononuclear cells.

No significant pathological changes could be detected in the control group at 112 dpi (Figure 7a,c,g). In the infected group, the previously described interstitial nephritis with tubular cell necrosis was also observed at 112 dpi (10/13 birds). Ureteral branches were characterized by excessive amounts of mucous in the lumen, and the lamina propria was expanded due to massive mononuclear cell infiltrations (Figure 7b). The ovary showed necrosis and sloughing of the ovarian surface epithelium (2/13 birds). The ovarian cortical stroma was infiltrated either by multi-focal heterophils with degranulation or focal mononuclear cells along with congestion of some blood capillaries (4/13 birds) (Figure 7d,e). A high rate of follicular atresia, mainly of obliterative type, was observed in infected group compared to control group. The later lesion was characterized by degeneration of small follicles with invasion of either granulosa cells or theca interna cells into the follicular lumen leading to destruction of the ovum (Figure 7f). In the oviducts that displayed no gross cystic lesions, both cranial and caudal portions were characterized by patchy deciliation and epithelial necrosis with desquamation. The submucosal connective tissue was characterized by edema, congested blood vessel, and focal mononuclear cell infiltrations (Figure 7h). The oviducts with grossly visible cysts showed multiple glands suffered from marked cystic dilatation with flattening of their epithelial lining, and their walls were thinner. Moreover, epithelial cell necrosis and sloughing with ciliary loss could be observed, and the lamina propria was identified by edema, congested blood vessels and mononuclear infiltrations (Figure 7i,j).

## 4. Discussion

The prevalence of the DMV/1639 IBV strain has increased significantly in Eastern Canada in recent years [30,31], which has been correlated to cystic oviduct and other reproductive tract anomalies observed in the field [32,33]. A long-term study was performed to examine the pathogenicity of the Canadian DMV/1639 strain to the reproductive tract of chickens infected early in their life. Following a challenge at 1 day of age, the DMV/1639 strain showed tropism to the respiratory, urogenital, and alimentary tracts. Significant gross and microscopic changes were observed in the respiratory and renal systems of young chickens. Significant cystic lesions were observed in the oviduct of the growing pullets that would lead to false layers. Additionally, the infective virus persisted for 16 weeks in the cecal tonsils with continuous cloacal viral shedding.

Early after infection, clinical signs and pathological changes, indicative of a respiratory infection followed by a renal disease, induced by the Canadian DMV/1639 strain, were consistent with most IBV strains that have been shown to cause cystic oviduct lesions [26,27,40,41]. However, the severity of the gross and histopathological lesions in the kidney were more prominent compared to the respiratory tract lesions. DMV/1639 strain was firstly isolated from NIB outbreak and showed high incidence of interstitial nephritis in the challenged birds [18]. Interestingly, the ability of IBV strains causing cystic oviduct, such as Australian T [25], QX-like strains [26], YN strain [40], TW I-type strains [41], and other variants [42] to replicate beyond the respiratory tract and cause characteristic nephropathogeincity has been described. In the current study, the presence of RNA and the detection of IBV antigen in all tissues collected from respiratory, renal, reproductive, and alimentary tracts indicated broad tissue tropism of the Canadian DMV/1639 strain. The IBV antigen has been commonly isolated from different target tissues between 5 and 10 dpi [4]. Hence, A 7-dpi time point was chosen to demonstrate the distribution of the virus to various tissues. There were no significant differences in the IBV genome loads between tissues collected at 7 dpi. This is probably due to the early dissemination of the virus to tissues beyond the respiratory tract. IBV detection in trachea, lung, kidney, ovary, oviduct, testis, and cecal tonsils was previously reported as early as 3 dpi [43].

A significantly higher cloacal compared to oropharyngeal viral shedding was recorded starting at 7 dpi, and this pattern persisted throughout the experiment. While the highest concentration of IBV can be detected in the trachea during the first 3–5 days post infection, the virus titer in the respiratory tract usually drops rapidly in the second week post infection [44]. Additionally, the roue of infection in our study was oculo-nasal with a fraction of the dose being swallowed giving possibility for early replication in the alimentary tract. Another explanation for the substantially higher cloacal viral shedding could be due to the infecting IBV strain. For example, the viral loads in cloacal swabs were higher compared to loads in tears between 2 to 14 dpi in IBV CalEnt-infected chickens; however, an opposite trend was observed in M41-challenged birds [45]. Similarly, 1-day-old SPF chickens infected with TW I-type IBV strain showed higher viral genome loads in the cloacal compared to oral swabs at 7, 14, and 156 dpi [41].

In the present study, the cut-off level of the average ELISA titer was reached at 35 dpi and demonstrated a significant difference from the control group at 91 dpi. Similarly, infection of 1-day-old SPF chicks with seven different IBV strains of QX, Mass, and 793/B types induced positive ELISA titers between 21 and 42 dpi [26]. The observed absence or weak antibody response upon infection at 1-day of age is likely to be due to the incomplete development of the chicken immune organs at hatch [46]. However, the delayed higher response detected at 91 dpi can be explained by the continuous excretion of the virus in the feces and possible reinfection. In a study that investigated IBV re-excretion following cyclosporin treatment, IBV-specific IgM was detected in the sera of birds 87 days after initial infection [47]. In our study, the possibility of reinfection can also be inferred by the frequent detection of the virus in the oropharyngeal swabs at different time points.

Long-term IBV persistence, particularly in the cecal tonsils and feces, has been previously reported [48,49]. In the current study, the highest IBV genome loads were detected in the cecal tonsils (11/13 infected birds) 16 weeks after initial exposure. Only 2–5 birds showed positive genome titers in the kidney, ovary, and oviduct. Similarly, Naqi et al. were able to isolate the virus from the internal organs between 27 and 154 dpi [49]. At the end of our experiment (112 dpi), although no obvious gross lesions were observed in the kidney, interstitial nephritis with massive mononuclear cell infiltrations were consistent microscopical findings. Chronic nephritis at 30 weeks after infection at 1-day of age was previously confirmed by virus isolation from birds exhibiting marked kidney lesions [20]. In older birds, either chronic virus infection or reoccurrence of an acute infection were the suggested mechanisms for the IBV induced kidney disease and death [48]. Our interesting finding of multi-focal heterophilic infiltrations in the ovary (Figure 7e) supports the reoccurrence of acute infections in the persistently infected birds. A similar finding of intense heterophilic infiltration in the ovary was observed 4 to 7 days post infection with a Mass type strain of IBV [50].

A high incidence of oviduct cysts that contributes to the development of false layer syndrome was the most interesting finding of this study. Approximately 46% of the inoculated birds developed cystic formations of varying sizes in the oviduct. This result coincides with the field observations of flocks with false layers peaked more than 50% lower than the expected level of egg production [32]. The observed gross and microscopic lesions in the oviduct were consistent with the early studies which investigated the damaging effects of Mass type IBV strains on the immature oviduct [24,51]. There was no significant difference in the lengths of oviducts between the infected and control groups at 112 dpi. However, reduced oviduct length was previously reported at different time points following IBV infection of young chickens [40]. The ovary lesions observed in this study included infiltration of heterophils and mononuclear cells, necrosis and sloughing of the ovarian surface epithelium, and, interestingly, higher rate of follicular atresia. The studies that investigated the pathogenicity of the IBV to the gonads of young birds were not consistent regarding the ovary. These discrepancies have even been described between strains of the same genotype. Various Mass type and QX-like strains have been shown to cause no substantial microscopic ovarian lesions [24,25,52]. On the other hand, lesions similar to our findings in the ovary have been described following infection with other IBV strains belong to Mass and QX genotypes [40,50]. The differences in the observations might be due to the difference in the sampling time. Nevertheless, our findings suggest that, in addition to the characteristic oviduct anomalies, the Canadian DMV/1639 strain may potentially impair laying performance by impacting the ovary.

Overall, our study confirms that the Canadian DMV/1639 strain is a highly pathogenic IBV strain that causes respiratory, renal, and reproductive pathologies.

## Figures and Tables

**Table 1:** Comparison of the cystic lesions in the oviduct between the infected and control groups at 16 weeks of age following infection at 1-day of age.

Group	Number of Chickens	Lesion Score (Cystic Formation) in Oviduct ^A^			
		−	+	++	+++
Infected	13	7	1	4 *	1
Control	15	15 *	0	0	0

^A^ Four-grade-scoring: negative (no cyst), moderate (cyst covering less than 20% of oviduct; +), marked (cyst covering 20% to 50%; ++), and severe (cyst covering more than 50% of oviduct; +++). * Significantly different (*p* < 0.05). The frequencies of lesions on oviduct were compared with the Fisher’s exact test.

## Data Availability

The datasets used and/or analyzed within the frame of the study can be provided by the corresponding author upon reasonable request.
